# Enhanced endogenous bone morphogenetic protein signaling protects against bleomycin induced pulmonary fibrosis

**DOI:** 10.1186/s12931-015-0202-x

**Published:** 2015-03-15

**Authors:** Ellen De Langhe, Frederic Cailotto, Vanessa De Vooght, Carolina Aznar-Lopez, Jeroen Alfons Vanoirbeek, Frank Prosper Luyten, Rik Jozef Urbain Lories

**Affiliations:** Laboratory of Tissue Homeostasis and Disease, Skeletal Biology and Engineering Research Center, Department of Development and Regeneration, KU Leuven, Herestraat 49, 3000 Leuven, Belgium; Experimental Toxicology Unit, Department of Public Health, KU Leuven, Herestraat 49, 3000 Leuven, Belgium; Skeletal Biology and Engineering Research Center, Department of Development and Regeneration, KU Leuven, Herestraat 49, 3000 Leuven, Belgium

**Keywords:** Transforming growth factor beta, Bone morphogenetic proteins, Bleomycin, Pulmonary fibrosis

## Abstract

**Background:**

Effective treatments for fibrotic diseases such as idiopathic pulmonary fibrosis are largely lacking. Transforming growth factor beta (TGFβ) plays a central role in the pathophysiology of fibrosis. We hypothesized that bone morphogenetic proteins (BMP), another family within the TGFβ superfamily of growth factors, modulate fibrogenesis driven by TGFβ. We therefore studied the role of endogenous BMP signaling in bleomycin induced lung fibrosis.

**Methods:**

Lung fibrosis was induced in wild-type or noggin haploinsufficient (*Nog*^*+/LacZ*^) mice by intratracheal instillation of bleomycin, or phosphate buffered saline as a control. Invasive pulmonary function tests were performed using the flexiVent® SCIREQ system. The mice were sacrificed and lung tissue was collected for analysis using histopathology, collagen quantification, immunohistochemistry and gene expression analysis.

**Results:**

*Nog*^*+/LacZ*^ mice are a known model of increased BMP signaling and were partially protected from bleomycin-induced lung fibrosis with reduced Ashcroft score, reduced collagen content and preservation of pulmonary compliance. In bleomycin-induced lung fibrosis, TGFβ and BMP signaling followed an inverse course, with dynamic activation of TGFβ signaling and repression of BMP signaling activity.

**Conclusions:**

Upon bleomycin exposure, active BMP signaling is decreased. Derepression of BMP signaling in *Nog*^*+/LacZ*^ mice protects against bleomycin-induced pulmonary fibrosis. Modulating the balance between BMP and TGFβ, in particular increasing endogenous BMP signals, may therefore be a therapeutic target in fibrotic lung disease.

## Background

Fibrotic diseases, including idiopathic pulmonary fibrosis (IPF), liver cirrhosis, diabetic nephropathy, arteriosclerosis, and systemic sclerosis (SSc) are a group of disorders for which effective therapy is largely lacking. Fibrosis is characterized by excessive production, deposition, and contraction of extracellular matrix and is considered an end-stage process responsible for organ dysfunction and potential organ failure [1]. Pulmonary fibrosis, idiopathic, in patients with systemic sclerosis or post-radiation or chemotherapy, is a major cause of death and its treatment is considered a major unmet need [2]. Many open questions remain with regards to the etiology and pathophysiology of fibrosis. Tissue fibrosis is generally considered a process of uncontrolled wound healing. Injury leads to activation, proliferation and migration of mesenchymal cells. A deregulated and unrestrained fibroblast compartment produces and deposits increased amounts of extracellular matrix (ECM), resulting in progressive fibrosis of skin and internal organs, leading to architectural damage. Normal wound healing is regulated by a complex set of interactions within a network of pro-fibrotic and anti-fibrotic cytokines and secreted proteins.

TGFβ is considered the major pro-fibrotic cytokine, orchestrating fibroblast function as a chemotactic factor [3,4], and promoting proliferation [5], the deposition of extracellular matrix proteins and fibroblast-to-myofibroblast conversion [6]. Active TGFβ signaling characterizes both idiopathic and connective tissue disease-associated lung fibrosis [7,8]. In rodents, adenoviral overexpression of TGFβ induces severe lung fibrosis [9,10]. The bone morphogenetic protein (BMP) family also belongs to the TGFβ superfamily. Originally discovered as molecules that induce ectopic bone formation in vivo [11], BMPs much alike TGFβs are now considered growth and differentiation factors that play key roles from early development to postnatal homeostasis, disease and repair of multiple organs, by regulating cell proliferation, lineage specification, differentiation, cell motility, and death [12]. Increasing evidence suggests a role for BMP signaling in postnatal pulmonary fibrotic disease such as idiopathic pulmonary fibrosis and SSc-related lung fibrosis [13]. The role of BMP antagonist gremlin has been highlighted, with increased expression in IPF [14] and transient pulmonary fibrosis in rats resulting from adenoviral *gremlin* overexpression [15]. Anti-fibrotic properties have been attributed to BMP4 and BMP7 in vitro, antagonizing effects of TGFβ on lung fibroblasts, suggesting the relevance of the TGF/BMP interplay and balance [16]. The role of endogenous BMPs in this context remains unknown.

In the BMP/TGFβ signaling cascades ligand-dependent receptor activation results in phosphorylation of SMAD family member (SMAD) proteins. Receptor-(R)-SMAD1, −5 and −8 are classically associated with the BMP cascade. R-SMAD2 and −3 are type I TGFβ receptor substrates, restricted to TGFβ signaling but TGFβs can also activate R-SMAD1, −5 and −8 [17]. The common-mediator SMAD4 (Co-SMAD4) will associate with R-SMADs upon their phosphorylation and the complex translocates to the nucleus. Transcription is regulated by the interaction of DNA-binding SMADs with transcriptional co-activators or co-repressors. Inhibitory SMAD6 and SMAD7 (I-SMADs) inhibit BMP and TGFβ signaling as they bind to the type I receptors, thus interfering with recruitment and subsequent phosphorylation of R-SMADs. Furthermore, SMAD6 interferes with the heterodimerization of the BMP-associated R-SMADs with SMAD4, serving as a Co-SMAD decoy for activated SMADs, thereby more selectively inhibiting BMP signaling [12].

Direct inhibition of growth factor signaling pathways has not translated well into a clinical setting [18] and may carry the risk of negatively influencing homeostatic processes in other tissues and organs. Modulation of endogenous regulators of pro-fibrotic signaling may represent an alternative approach. Here, we studied the role of endogenous BMP signaling in lung and skin fibrosis using bleomycin-induced mouse models of fibrosis in wild-type (WT) and noggin (*Nog*) haploinsufficient (*Nog*^*+/LacZ*^) mice. NOG is a secreted extracellular BMP antagonist that binds different BMPs thereby interfering with receptor activation [19]. *Nog*^*+/LacZ*^ mice serve as a model of derepressed BMP signaling [20,21].

In this work, we provide new evidence for a critical endogenous balance between the related TGFβ and BMP pathways, determining in vivo fibrotic outcomes in the lung.

## Methods

### Animals

Eight-week old male *Nog* heterozygous mice (*Nog*^*+/LacZ*^) on a C57BL/6 background (a gift from R. Harland, Berkeley, CA), weighing 22–25 grams, were used [22,23]. Wild-type (WT) littermates served as controls. Mice were genotyped by polymerase chain reaction (PCR) genomic analysis for *LacZ* [20]. The KU Leuven Ethical Committee for animal research approved all experiments.

### Bleomycin-induced pulmonary fibrosis

Lung fibrosis was induced by intratracheal instillation of 0.05U bleomycin (BLM) (Sanofi-Aventis, Diegem, Belgium), dissolved in 50 μl of sterile phosphate buffered saline (PBS), or PBS as a control. An incision was made in the shaved anterior neck region. Blunt dissection of the salivary glands and the pretracheal muscles along the midline exposed the trachea. The animal was placed in 70° upright position. A 0.3 ml syringe with a 30G needle was placed between two tracheal cartilaginous rings after which BLM or PBS was slowly instilled. The wound was closed with Vicryl 5.0. Pulmonary fibrosis was induced in WT or *Nog*^+/LacZ^ mice (WT: BLM n = 28, PBS n = 17; *Nog*^+/LacZ^: BLM n = 14, PBS n = 5). 4 weeks after baseline induction, invasive pulmonary function tests were performed using the flexiVent® SCIREQ system (SCIREQ, Montreal, Canada). Mice were subsequently sacrificed and pulmonary tissue was collected for histopathology, collagen quantification, immunohistochemistry and gene expression analysis. For the timecourse experiment, BLM was administered and mice were sacrificed 1, 3, 7, 14 and 21 days after induction.

### Pulmonary function tests

Mice were anesthetized with an intraperitoneal injection of pentobarbital (70 mg/kg) (CEVA, Brussels, Belgium) to suppress spontaneous breathing. After a tracheostomy, the mice were connected to the flexiVent system (SCIREQ). The computer-controlled small animal instrument ventilated the mice quasi-sinusoidally with a tidal volume of 10 ml/kg at a frequency of 150 breaths/minute and a positive end-expiratory pressure of 2 cmH_2_O to achieve a mean lung volume close to that during spontaneous breathing. On the flexiVent we performed a Snapshot perturbation. Each time before performing this perturbation, a total lung capacity perturbation (TLC) was carried out to normalize the lungs. The data from the TLC perturbation were not used. The snapshot perturbation was performed until three acceptable measurements (coefficient of determination > 0.95) were recorded in each individual subject, of which an average was calculated. The snapshot perturbation was imposed to measure resistance (R), compliance (C), and elastance (E) of the whole respiratory system (airways, lung, and chest wall). Only the data of the C are presented in the results [24].

### Histological analysis

After completion of invasive pulmonary function tests, mice were euthanized with pentobarbital overdose. The tracheal cannula was removed, the chest cavity was opened and heart and lungs were removed en bloc. The left lung was collected for histopathology, inflated with 400 μl of 10% formalin/PBS via the left main bronchus and fixed in formalin for 24 hours. After paraffin embedding, 5 μm sections were cut throughout the whole lung. Five sections, with 1 mm interval, were stained with hematoxylin-eosin (H&E). The semiquantitative Ashcroft score was used to score pulmonary fibrosis [25]. In short, upon 100x magnification, each successive field was given a score ranging from 0 (normal lung) to 8 (total fibrous obliteration of the field). All scores from 5 sections were averaged.

### Hydroxyproline assay

The right lung lobes were collected and stored at −80°C for later analysis. Hydroxyproline quantification was performed as described [26]. Right lung lobes were hydrolyzed for 3 hours in 6 M HCl at 120°C. After cooling down for 15 minutes, pH was neutralized (pH 6–7) with 1 N NaOH. Samples were diluted 1/20 in sterile H_2_0. Free hydroxyproline was oxidized with Chloramine-T for 20 minutes after which the oxidation reaction was stopped using 70% perchloric acid. Ehrlich’s reagent was added and samples heated for 20 minutes in a 60° water bath. After cooling down for 5 minutes, absorbance was measured at 570 nm and concentrations were calculated based on a standard curve.

### Immunohistochemistry

For phosphorylated SMAD1/5/8 (pSMAD1/5/8) immunohistochemistry, sections were quenched with 3% H_2_O_2_/H_2_O. Antigen retrieval was performed in a 10 mM sodium citrate buffer and sections were preincubated with donkey serum (20% in Tris buffered saline (TBS)). Sections were incubated overnight at 4°C with primary antibody against pSMAD1/5/8 (1:50 dilution; Cell Signaling, Leiden, The Netherlands). Negative control studies were performed with species-specific IgG (Jackson ImmunoResearch, Te Huissen, The Netherlands). Secondary antibodies were horseradish peroxidase (HRP)–conjugated antibodies (1:200 dilution; Jackson ImmunoResearch, Te Huissen, The Netherlands). For proliferating cell nuclear antigen (PCNA) immunohistochemistry, sections were quenched with 3% H_2_O_2_/H_2_O. Antigen retrieval was performed in a 10 mM sodium citrate buffer and sections were preincubated with goat serum (20% in Phosphate buffered saline (PBS)-Tween). Sections were incubated overnight at 4°C with primary antibody against PCNA (1:500 dilution; Abcam ab2426, Cambridge, United Kingdom). Negative control studies were performed with IgG and PBS. Secondary antibodies were horseradish peroxidase (HRP)–conjugated antibodies (1:100 dilution; Jackson ImmunoResearch, Te Huissen, The Netherlands). TUNEL stainings were performed using the In Situ Cell Death Detection kit, TMR Red according to the protocol (Roche, Vilvoorde, Belgium).

### Western blot analysis

Lung tissues were homogenized in 200 μl Cell Extraction Buffer (Life Technologies, Gent, Belgium) supplemented with 5% Proteinase Inhibitor Cocktail (Sigma Aldrich, Diegem, Belgium) and 1 mM phenylmethylsulfonyl fluoride (PMSF) (Sigma Aldrich, Diegem, Belgium). 5 μg of protein were loaded onto a 4–12% Bis-Tris gel (Life Technologies, Gent, Belgium). Electrophoresis was carried out in running buffer (NuPAGE MOPS SDS running buffer (20×); Life Technologies, Gent, Belgium) at 200 V for 30 minutes. Proteins were transferred onto a polyvinylidene difluoride membrane using semi-dry transfer (Bio-Rad, Temse, Belgium) for 60 minutes. Nonspecific binding sites were blocked for 1 hour with 5% skimmed milk powder in TBS–Tween (TBST). Blots were probed with rabbit antiserum against total and phosphorylated forms of SMAD1/5/8 or SMAD2/3 (dilution 1/1000 in 5% Bovine Serum Albumin (BSA)) overnight at 4°C. Next day, the membrane was incubated with a HRP–conjugated goat anti-rabbit secondary antibody (dilution 1/5000 in 5% skimmed milk powder in TBST) (Jackson ImmunoResearch, Te Huissen, The Netherlands) for 1 hour. All washes were performed with TBST (3 × 10 minutes).

### RNA isolation and quantitative reverse transcription-polymerase chain reaction

Total RNA was extracted from lung homogenates using NucleoSpin RNA isolation kit (Machery Nagel, Eupen, Belgium) and reverse-transcribed using First strand cDNA synthesis kit (Fermentas, St. Leon-Rot, Germany). Gene expression levels were quantified using Taqman Assays-on-Demand (Life Technologies, Gent, Belgium). Expression was normalized to hypoxanthine-guanine phosphoribosyltransferase (*Hprt*) and subsequently normalized to the control condition using the comparative cycle threshold method (ΔΔCT).

### Statistics

Data were analyzed using GraphPad Prism 6.0 (www.graphpad.com). For the in vivo experiments and ex vivo analyses, two-way ANOVA was used after rank transformation to control for differences in variance. Results are reported for main effects (genotype, BLM vs. PBS) and for interaction. For two-group comparisons unpaired t-tests applying Welch correction for unequal variances when necessary were used. P-values < 0.05 were considered significant.

## Results

### Noggin haploinsufficiency protects mice from lung fibrosis

**Figure 1:**
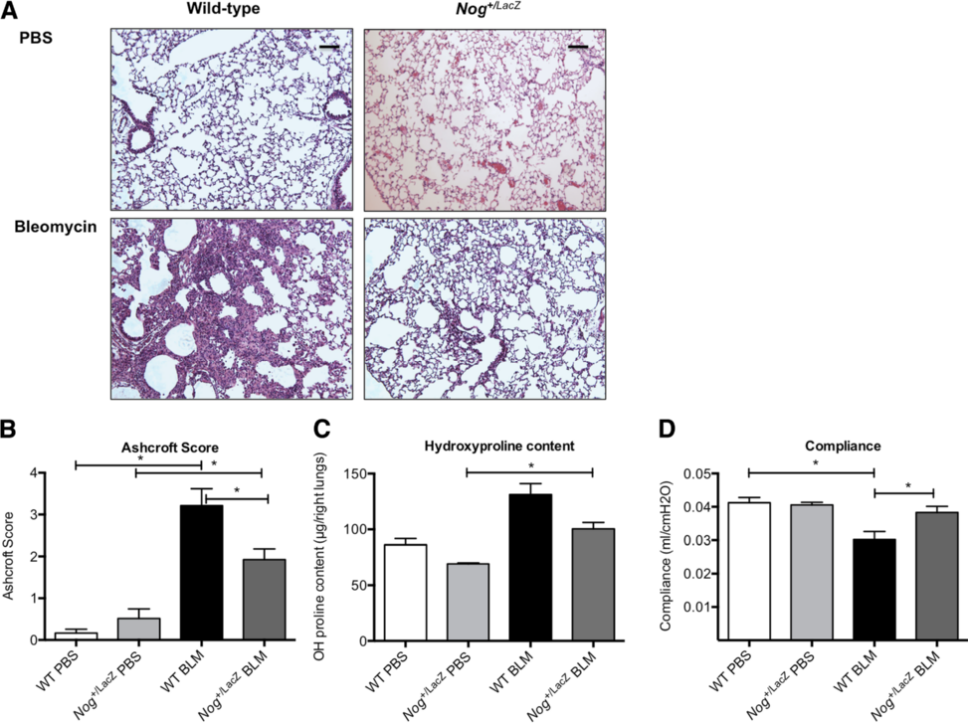
**Noggin haploinsufficiency protects mice from bleomycin-induced lung fibrosis. (A)** Representative images of lungs from WT and *Nog* ^*+/LacZ*^ mice, 4 weeks after PBS or bleomycin instillation. (Hematoxylin-Eosin staining; bar = 200 μm) **(B)** Ashcroft score from WT mice (PBS n = 20, BLM n = 25) and *Nog* ^*+/LacZ*^ mice (PBS n = 5, BLM n = 14), 4 weeks after bleomycin instillation. **(C)** Hydroxyproline content of right lungs from WT mice (PBS n = 5, BLM n = 5) and *Nog* ^*+/LacZ*^ mice (PBS n = 3, BLM n = 8), 4 weeks after bleomycin instillation. **(D)** Pulmonary compliance of WT mice (PBS n = 12, BLM n = 16) and *Nog* ^*+/LacZ*^ mice (PBS n = 7, BLM n = 10), 4 weeks after bleomycin instillation. (*p < 0.05 by Sidak multiple comparison test post 2-way ANOVA; data presented as mean and SEM).

*Nog*^*+/LacZ*^ mice were previously established as a model of enhanced BMP signaling [20,21,27]. The lungs of these mice appeared healthy with normal lung histology (Figure 1A) and compliance (0.0412 +/− 0.0016 ml/cm H_2_O (mean & SEM) in wild-type (WT) versus 0.0406 +/− 0.0008 ml/cm H_2_O (mean & SEM) in *Nog*^*+/LacZ*^ mice). In the bleomycin-induced model, *Nog*^*+/LacZ*^ mice were partially protected from lung fibrosis compared to WT mice (Figure 1A). Histopathological Ashcroft score was significantly different as indicated by 2-way ANOVA analysis (p = 0.0203 for interaction between genotype and bleomycin treatment, p < 0.0001 for main effect bleomycin and p = 0.794 for main effect genotype) (Figure 1B). Similarly *Nog*^*+/LacZ*^ mice had lower collagen content (Figure 1C) (p = 0.9 for interaction between genotype and bleomycin treatment, p < 0.0001 for main effect bleomycin and p = 0.013 for main effect genotype). Pulmonary compliance was significantly different as indicated by 2-factor analysis (p = 0.0431 for interaction between genotype and bleomycin treatment, p = 0.0002 for main effect bleomycin and p = 0.283 for main effect genotype) (Figure 1D).

### TGFβ and BMP signaling show inversed dynamics in the bleomycin lung fibrosis model

**Figure 2:**
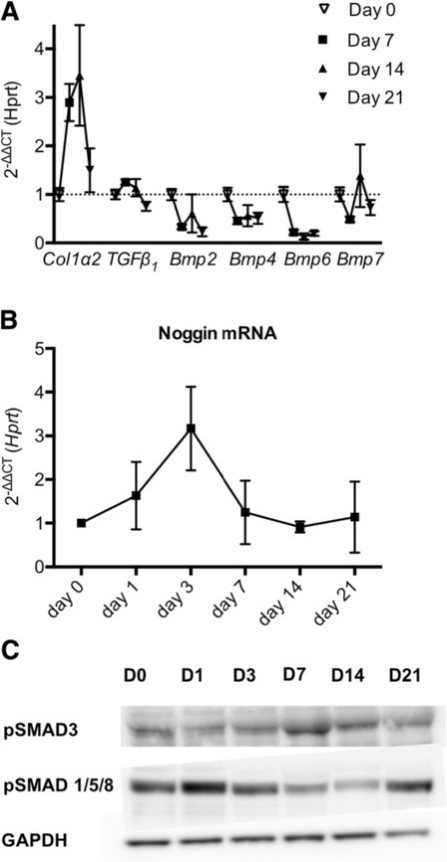
**Dynamics of TGFβ and BMP signaling of wild-type mice in bleomycin-induced lung fibrosis. (A)** Gene expression level of *TGFβ*_*1*_ *, Collagen1α2*, *Bmp2*, *Bmp4*, *Bmp6* and *Bmp7* (n = 5 at day 0, n = 4 at day 7 and 14, n = 2 at day 21, data are mean and SEM of ΔΔCT values normalized to control conditions and *Hprt*). **(B)** Gene expression level of *noggin* (n = 3 at day 0 and day 1, n = 4 at day 7 and 14, n = 2 at day 21, data are mean and SEM of ΔΔCT values normalized to control conditions and *Hprt*). **(C)** Western blot of representative lung protein extracts from mice 1, 3, 7, 14 and 21 days after bleomycin instillation, labeled with antibodies against pSMAD3, pSMAD1/5/8 and GAPDH.

Gene expression analysis showed dynamic changes in TGFβ/BMP signaling in the course of bleomycin-induced lung fibrosis. *Tgfβ*_*1*_ mRNA is transiently upregulated at day 7 and 14 after bleomycin instillation (Figure 2A), paralleling expression of *Coll1α2* (Figure 2A), returning to baseline levels by day 21. In contrast, expression levels of *Bmp2, −4, −6* but not *Bmp7* were downregulated over the course of the model (Figure 2A). *Nog* expression was detected in the lung, and was upregulated early after bleomycin instillation, peaking at day 3 (Figure 2B). Western Blot analysis confirmed the downstream effect of downregulated BMP ligands and upregulated BMP antagonist noggin, showing decreased pSMAD1/5/8 levels while the TGFβ pathway showed an inverse activated course, reflected by transient increased in pSMAD3 (day 7–14) (Figure 2C).

**Figure 3:**
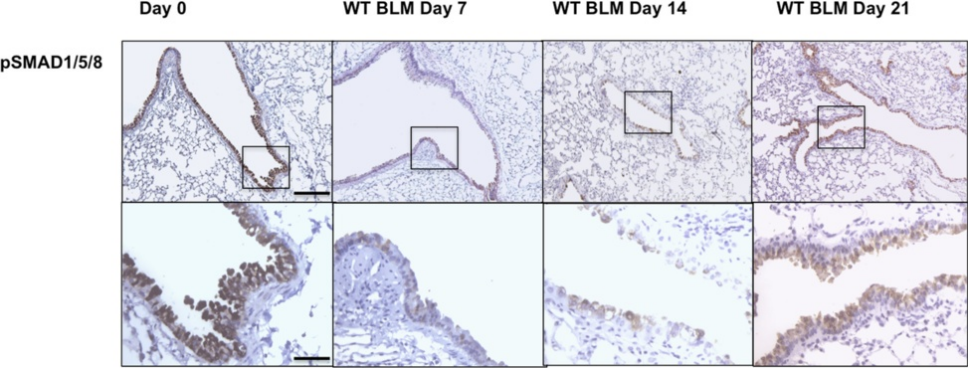
**Dynamics of TGFβ and BMP signaling of wild-type mice in bleomycin-induced lung fibrosis.** pSMAD1/5/8 immunohistochemistry at different time points in the bleomycin model in WT mice. (bars are 500 μm and 125 μm respectively).

Immunohistochemistry further supported this dynamic decrease in phosphorylation of SMAD1/5/8 during the course of the model. PSMAD1/5/8 signal was high in bronchial epithelial cells in healthy 8-week old mice (Figure 3). No positive cells were identified in more distal airways, alveoli or blood vessels. Upon bleomycin challenge, the number and intensity of pSMAD1/5/8 positive cells was reduced by day 7, resulting in only scattered, faint staining in bronchial epithelial cells, with almost absent nuclear staining. The number of pSMAD1/5/8 positive cells remained low 14 days after bleomycin instillation, and slowly recovered by day 21.

**Figure 4:**
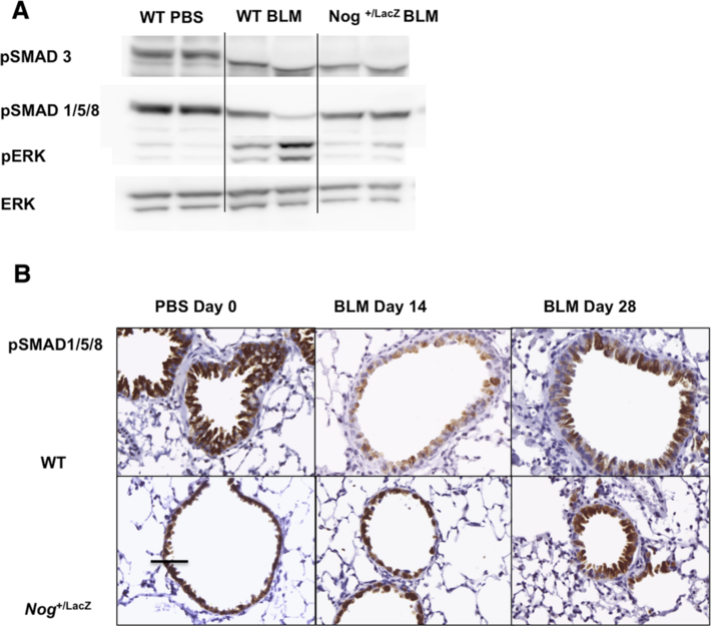
**Dynamics of TGFβ and BMP signaling of** ***Nog*** ^***+/LacZ***^ **mice in bleomycin-induced lung fibrosis. (A)** Western blot of representative lung protein extracts from WT and *Nog* ^*+/LacZ*^ mice, 4 weeks after treatment with bleomycin or PBS, labeled with antibodies against pSMAD3, pSMAD1/5/8, ERK and pERK. **(B)** pSMAD1/5/8 immunohistochemistry at different time points in PBS or bleomycin treated WT or *Nog* ^*+/LacZ*^ mice (bar = 150 μm).

**Figure 5:**
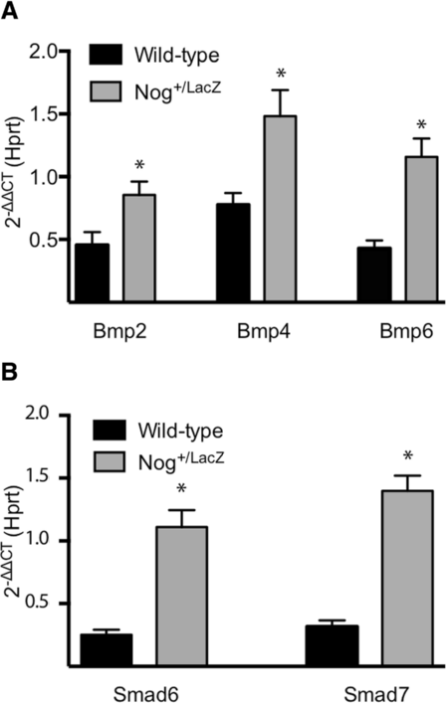
**Dynamics of TGFβ and BMP signaling of** ***Nog*** ^***+/LacZ***^ **mice in bleomycin-induced lung fibrosis. (A)** Gene expression level of *Bmp’s in* WT and *Nog* ^*+/LacZ*^ mice, 4 weeks after treatment with bleomycin (n = 6-10 per group; data presented as mean and SEM). **(B)** Gene expression level of *Smad6* and *Smad7* in WT and *Nog* ^*+/LacZ*^ mice, 4 weeks after treatment with bleomycin (*p < 0.05; n = 5 per group; data presented as mean and SEM values normalized to control conditions and *Hprt*).

The changes in the BMP signaling pathway listed above were attenuated in *Nog*^*+/LacZ*^ mice. Levels of pSMAD1/5/8 and pSMAD3 appeared unaffected when compared to PBS treatment (Figure 4A). Levels of pSMAD1/5/8 inversely correlated with activation of ERK, a mitogen activated protein kinase associated with progression of lung fibrosis [28]. Immunohistochemistry confirmed the sustained activity of the BMP signal after bleomycin instillation, reflected by the persistence of pSMAD1/5/8 positivity in bronchial epithelial cells in *Nog*^*+/LacZ*^ mice, 14 days after bleomycin instillation (Figure 4B). Furthermore, in *Nog*^*+/LacZ*^ mice *Bmp2*, −*4* and −*6* levels were higher than in WT mice (Figure 5A). In addition, expression levels of inhibitory SMADs were enhanced as compared to WT mice suggesting homeostatic regulation of TGFβ signaling (Figure 5B).

### Evidence for reduced apoptosis and reduced proliferation in *Nog*^*+/LacZ*^ mice after bleomycin instillation

**Figure 6:**
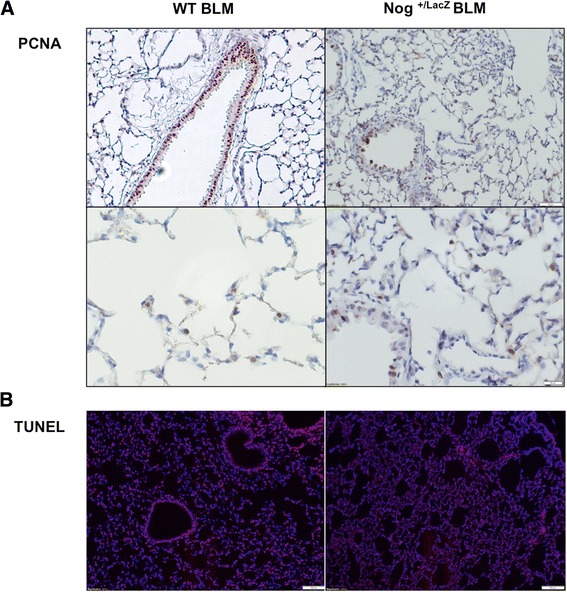
**Evidence for reduced apoptosis and reduced proliferation in** ***Nog*** ^***+/LacZ***^ **mice after bleomycin instillation. (A)** PCNA immunohistochemistry in WT and *Nog* ^*+/LacZ*^ mice, 4 weeks after treatment with bleomycin. **(B)** TUNEL in WT and *Nog* ^*+/LacZ*^ mice, 4 weeks after treatment with bleomycin.

We evaluated proliferation and apoptosis in WT and *Nog*^*+/LacZ*^ mice, 4 weeks after bleomycin instillation. We observed higher numbers of PCNA positive cells in WT mice compared to *Nog*^*+/LacZ*^ mice (Figure 6A). This was most evident in bronchial epithelial cells. We assessed apoptosis using TUNEL staining and demonstrate that apoptosis is seen both in bronchial and alveolar cells in WT mice, but is largely absent in *Nog*^*+/LacZ*^ mice (Figure 6B).

## Discussion

Fibrotic diseases, like systemic sclerosis-associated lung fibrosis and idiopathic pulmonary fibrosis are severe diseases with high morbidity and mortality. TGFβ superfamily members are crucial in lung development. Increasing evidence supports an additional role for these pathways in postnatal homeostasis and disease as they regulate mesenchymal proliferation, myofibroblast differentiation, extracellular matrix production and fibrotic disease progression [29,30]. Here we show that changes in endogenous BMP signaling in noggin haploinsufficient mice reduce lung fibrosis in the bleomycin-induced model, suggesting that BMPs are homeostatic signals in the lung.

The noggin haploinsufficient mouse is a genetic model that results in de-repression of BMP signaling and highlights the impact of discrete shifts in BMP signaling due to the partial absence of the broad-spectrum endogenous antagonist noggin (about 50% reduction in expression). *Nog*^*+/LacZ*^ mice show a discrete skeletal phenotype with fusion of tarsal joints depending on the background strain [21], are partially protected against cartilage damage in models of osteoarthritis [20] and have conductive hearing loss [27]. We detected no differences as compared to wild-type animals in general histology of the lungs and in pulmonary function tests. Upon challenge with bleomycin, progression of fibrosis in the lungs was significantly reduced. Although we detected *Nog* expression in the mouse lung, increased systemic Noggin levels likely contribute to these effects as suggested earlier in arthritis models in this specific transgenic mouse strain [20].

The anti-fibrotic properties of exogenous BMPs have been investigated in different cells and organs. In a mouse model of pancreatic fibrosis, BMP signaling plays a protective role [31]. BMP2 antagonizes renal fibrosis in the rat ureter obstruction model in vivo [32] and promotes the catabolism of the type I TGFβ receptor in renal fibroblasts. In lung fibroblasts, BMP2 inhibits fibroblast-to-myofibroblast transdifferentiation in vitro through PKC-mediated inhibition of endothelin-dependent fibroblast chloride currents [33]. BMP4 and BMP7 attenuate TGFβ-induced activation of normal human lung fibroblasts, reducing proliferation, differentiation, collagen expression and the activity of proMMP1 and proMMP2 [16]. BMP7 opposes TGFβ_1_-mediated collagen induction in mouse pulmonary myofibroblasts [34] and inhibits silica-induced lung fibrosis in rats [35]. Interfering with BMP signaling appears to affect postnatal lung homeostasis. Tilorone, a BMP inducer, has been shown to have antifibrotic effects in a mouse model of silica-induced lung fibrosis [36]. Overexpression of the BMP antagonist *gremlin* results in transient fibrosis [15] associated with increased apoptosis, hyperplasia and proliferation of type I and type II pneumocytes and increased proliferation of fibroblasts. In IPF, the BMP antagonist gremlin was consistently shown to be upregulated and localized in mesenchymal cells [14]. These findings were elaborated in asbestos-induced murine lung fibrosis [37], with TGFβ type I receptor-dependent upregulation of gremlin expression at day 14 with interstitial localization. Consistent with our observations, a positive pSMAD1/5/8 signal was observed in bronchial epithelial cells of controls but was almost undetectable in asbestos-exposed epithelial cells. Using a BMP reporter mouse, Sountoulidis et al. demonstrate that BMP signaling is increased in alveolar epithelial cells following bleomycin injury and that this increase coincides with an enrichment of alveolar progenitor cells [30]. In our work, we demonstrate clear dynamic changes in the activity of the BMP signaling pathways in bronchial epithelial cells, in concordance with the findings of Myllarniemi et al. [37]. We do not detect pSMAD1/5/8 positive alveolar epithelial cells. Furthermore, we detect a downregulation of *Bmp2, 4* and *6* expression. It cannot be ruled out that this apparent paradox between our work and the work by Sountoulidis et al. results from methodological or intrinisic differences between the models. Smad phosphorylation is a rapid and short-lived event upon exposure to BMP signaling. Therefore the reporter approach with BMP responsive element driven gene expression may be more sensitive to document the range of BMP signaling. In contrast, immunohistochemistry may highlight the most dynamically regulated sites; in this case the bronchial epithelial cells. Sountoulidis et al. induce lung fibrosis in mice that are of older age, and use significantly lower bleomycin doses. Furthermore, it is known that TGFβ is able to result in phosphorylation of SMAD1/5/8 through Alk1, resulting in non-BMP driven activation of downstream pSMAD1/5/8 signaling [38–40].

TGFβ and BMP signaling are crucial in the epithelial-mesenchymal interactions [41] in lung morphogenesis that regulate branching, cell fate, proliferation and death of both mesenchymal and endodermal cells [42,43]. In our work, we evaluated cell fate after bleomycin instillation in WT and *Nog*^*+/LacZ*^ mice. We show that WT mice have more apoptosis in bronchial and alveolar epithelial cells and increased proliferation in bronchial epithelial cells, when compared to *Nog*^*+/LacZ*^ mice. We hypothesize that Noggin haploinsufficiency and increased BMP signaling activity results in less tissue remodeling with reduced cell proliferation and cell death.

Based on our data, we hypothesize that in healthy lungs, BMPs play a role in normal epithelial homeostasis. Upon bleomycin exposure, the integrity of the bronchial epithelial cells is threatened, with repression of the BMPs. The reduced availability of BMP ligands results in decreased BMP signaling activity and decreased antagonism of pro-fibrotic TGFβ signaling in the mesenchymal compartment.

## Conclusions

Alteration of the TGFβ/BMP balance in vivo, demonstrated in the *Nog*^*+/LacZ*^ mice, effectively alters fibrotic outcomes, demonstrating the biological relevance of these findings. The balance between BMPs and TGFβs may have therapeutic potential with BMP signaling potentiating drugs currently under research. The data presented here therefore could contribute towards more focus on endogenous and exogenous BMP modulators as potential therapeutics for these devastating fibrotic diseases.

## Abbreviations

ΔΔCT: Comparative cycle threshold
BLM: Bleomycin
BMP: Bone morphogenetic protein
BSA: Bovine Serum Albumin
C: Compliance
Co-SMAD4: Common-mediator SMAD4
E: Elastance
ECM: Extracellular matrix
H&E: Hematoxylin-eosin
*Hprt*: Hypoxanthine-guanine phosphoribosyltransferase
HRP: Horseradish peroxidase
I-SMADs: Inhibitory SMADs
IPF: Idiopathic pulmonary fibrosis
Nog: Noggin
*Nog*^*+/LacZ*^: Noggin haploinsufficient
PBS: Phosphate buffered saline
PCR: Polymerase chain reaction
PMSF: Hhenylmethylsulfonyl fluoride
R-SMAD: Receptor-associated SMAD
R: Resistance
SSc: Systemic sclerosis
TBS: Tris buffered saline
TBST: TBS–Tween
TGFβ: Transforming growth factor beta
TLC: Total lung capacity
WT: Wild-type
